# Effect of sharing health messages on antenatal care behavior among women involved in microfinance-based self-help groups in Bihar India

**DOI:** 10.1186/s41256-020-0132-0

**Published:** 2020-02-07

**Authors:** Monika Walia, Laili Irani, Indrajit Chaudhuri, Yamini Atmavilas, Niranjan Saggurti

**Affiliations:** 1 Population Council, Zone 5A, Ground Floor India Habitat Centre, Lodi Road, New Delhi, Delhi 110003 India; 2 Project Concern International, 38, Okhla Phase 3 Rd, Okhla Phase III, Okhla Industrial Area, New Delhi, Delhi 110020 India; 3 Bill & Melinda Gates Foundation, Capital Court, 5th Floor, Olof Palme Marg, Munirka, Delhi India

**Keywords:** Self-help group (SHG), Antenatal care (ANC), Propensity score matching (PSM), Health integration, Health messaging, ANC outcomes, Bihar India

## Abstract

**Background:**

Bihar state in India has one of the highest rates of maternal and infant mortality in South Asia. Microfinance-based self-help groups (SHGs), involving rural women, are being utilized to improve maternal and child health practice and reduce mortality. SHG members receive information on key maternal and child health practices as well as encouragement for their practice. This study measures the association of health messaging to SHG members with their antenatal care (ANC) behaviors.

**Methods:**

The study was conducted in eight districts of Bihar in 2016. A three-stage cluster sampling design (with a random selection of blocks, villages, and SHGs) selected the sample of 1204 SHG members who had an infant child; of these, 597 women were members of SHGs that received dedicated sessions on health messages, while 607 women belonged to SHGs that did not. To examine the impact of the health intervention on ANC practice, radius caliper method of propensity score matching controlled for various socio-demographic characteristics between the two groups.

**Results:**

Most of the interviewed women (91.5%) belonged to a scheduled caste or tribe. Nearly 44% of SHG members exposed to the health intervention were engaged in some occupation, compared to 35% of those not exposed to the intervention. After matching unexposed SHG women with exposed SHG women, no significant differences were found in their socio-demographic characteristics. Findings suggest that exposure to a health intervention is associated with increased likelihood of at least four ANC visits by SHG women (ATE = 7.2, 95% CI: 0.76–13.7, *p* < 0.05), consumption of iron-folic acid for at least 100 days (ATE = 8.7, 95% CI: 5.0–12.5, *p* < 0.001) and complete ANC (ATE = 3.6, 95% CI: 2.3–4.9, *p* < 0.001), when compared to women not exposed to the health intervention.

**Conclusions:**

The study shows that sharing health messages in microfinance-based SHGs is associated with significant increase in ANC practice. While the results suggest the potential of microfinance-based SHGs for improved maternal health services, the approach’s sustainability needs to be further examined.

## Background

India was unable to achieve its Millennium Development Goal 5, reduction of maternal mortality, primarily due to high rates of maternal mortality in states such as Bihar. According to the Sample Registration System 2011–2013, Bihar’s maternal mortality ratio (MMR) was estimated at 208 deaths out of 100,000 live births, higher than the national MMR of 167 deaths per 100,000 live births [1]. Reducing Bihar’s MMR will enable the nation to come closer to meeting its Sustainable Development Goal 3.1 of reducing its MMR to less than 70 per 100,000 live births by 2030.

The World Health Organization’s (WHO) Safe Motherhood Program identifies antenatal care (ANC) as one of four domains for prevention of maternal and infant mortality [2]. Several studies show that ANC provides opportunities for identifying pregnancies at risk and increasing safe deliveries [3, 4].

Recent data from India suggest that while 51.2% of women nationally make four or more ANC visits, only 14.4% of women in Bihar do [5]. In addition, only 3.3% of pregnant women in Bihar receive complete ANC (i.e. at least four ANC visits, at least one tetanus toxoid (TT) injection, and iron-folic acid (IFA) tablets or syrup for 100 days or more) [6]. Studies in similar contexts have identified reasons for lack of complete ANC, such as keeping pregnancy secret (for cultural reasons), lack of perceived ANC benefits, family member discouragement, direct and indirect costs, lack of transportation, inadequate infrastructure, distances to clinics, lack of accompaniment for ANC visits, and poor healthcare staff attitudes [4, 7].

Microfinance-based women’s collective organizations, called self-help groups (SHGs), provide a promising social medium through which messages to increase ANC coverage can be shared [8–10]. These messages are shared through a behavior change communication (BCC) approach, coupled with self-generated community mobilization among group members. A few studies have measured the effects on maternal and child health from community-based interventions implemented through women’s SHGs. The Makwanpur trial in Nepal implemented a participatory learning cycle through women’s SHGs that was followed by an 88% reduction in maternal mortality and a 30% reduction in neonatal mortality after 3 years [11]. Similar interventions in the Indian states of Jharkhand and Orissa found that infants born to SHG mothers had significantly improved chances of survival within the first 6 weeks of life compared to infants born to women who were not SHG members [12]. These studies, however, fail to measure effects of such interventions at scale, and with comparison groups as controls, impeding the scalability and sustainability of such interventions.

While past studies have looked at the social and economic impacts of microfinance-based SHGs, limited evidence is available on the potential of utilizing the existing and growing national and state government structures of microfinance-based SHGs. This article attempts to reduce deficiencies in the existing literature by analyzing the changes in ANC outcomes among women in Bihar who belong to an established microfinance-based program in which health, nutrition, and sanitation interventions are incorporated. The study employed a rigorous existing methodology and control group to compare the behaviors of women exposed to the health intervention with those not exposed to the health BCC intervention. Such a comparison will shed light on whether combining a health program with a microfinance-based SHG program, and taking it to scale, improves ANC practices and outcomes among the most marginalized populations, who are more often SHG members.

## Methods

### Study design and setting

This post-intervention study was conducted from June through August 2016, in eight intervention districts in Bihar, namely Patna, Saharsa, East Champaran, West Champaran, Samastipur, Begusarai, Gopalganj, and Khagaria. Located in the eastern part of India, Bihar is the country’s third most populous state, with a population of 104 million, approximately 9 % of the nation’s population. Nearly 89% of Bihar is rural, and 17% of household heads in Bihar belong to a scheduled caste or tribe (SC/ST) [13]. Nationally, Bihar has the highest population proportion in the lowest wealth quintile. Both neonatal and infant mortality rates in Bihar are higher than the national average [6].

Since 2006, the Government of Bihar has established SHGs for women, which are primarily microfinance-based institutions, popularly known as ‘JEEViKA’ [14]. Each SHG generally comprises 10 to 15 members, women who often belong to marginalized populations, i.e. SC/ST and poorest families. In 2015, JEEViKA worked with an implementing partner to introduce sessions on healthy maternal and child health practices within existing SHGs. The sessions on maternal and child health were conducted by health workers known as community mobilizers (CMs), who convene the SHG meetings. After training, CMs were expected to share messages on healthy maternal and child health practices once a month, in one of four weekly SHG meetings, over the course of a year, through participatory discussions, stories, and games. Eight discrete sessions were designed, with ANC discussed in four. In the sessions where ANC was discussed, women were advised to have four ANC checkups during pregnancy, to eat a balanced diet, receive necessary immunizations, and take IFA supplements. The aim of sharing this information was not only for pregnant women to learn about ANC behaviors, but to garner support from their fellow members to help their practice of those behaviors.

The groups of women in SHGs who were exposed to the health intervention were considered study cases, and those who were not part of the intervention (i.e. not provided with health BCC messaging) were considered the control group. The survey’s inclusion criteria called for women who were SHG members and who had given birth to a live infant during the year preceding the survey.

### Sample size and sampling method

A three-stage cluster sampling design selected the desired sample of SHG members. In the first stage, study blocks were randomly selected from two groups—blocks where the health BCC intervention was ongoing and blocks where the intervention was not present. In the second stage, villages were randomly selected from the selected blocks. In the third stage, SHGs were systematically selected from each of the selected villages. JEEViKA’s list of SHGs was used as a sampling frame for SHG selection. If a SHG had no members who had given birth in the year preceding the survey, the field team replaced the group with another SHG in the village. All eligible women from every selected SHG were interviewed, using a structured interview schedule. The sample for this research came from a larger pre-post quasi-experimental study that evaluated the effect of BCC interventions on newborn care practices among the most marginalized women in SHGs [15].

A total sample of 1204 SHG women was estimated to detect the differences between the case and control arms for three key outcomes of interest—at least four ANC visits, safe delivery, awareness of sanitation—with 95% confidence and 5% margin of error. This sample allowed inquiries about exposure to the intervention and ANC practices with minimum recall bias. Of the 1204 SHG women surveyed, 597 were from SHGs exposed to the health BCC intervention, residing in intervention blocks (SHG + H), and 607 belonged to SHGs not exposed to the health intervention, residing in non-intervention blocks (SHG only). The study design prevented contamination of the study arms.

### Data collection

All research investigators were female, with bachelor’s or master’s degrees in either psychology or social work. Investigators were trained to be sensitive to potential confidentiality issues. Research investigators attended SHG meetings and identified eligible married women. Research staff then spoke to eligible women about the survey. If a woman indicated interest in participating, a private location, often at the potential respondent’s home, or nearby, was identified for the consent process and subsequent interview. Face-to-face interviews were conducted by the trained, native investigators using a pre-coded questionnaire translated into the local language (Hindi). Women were asked about their knowledge, practice, and prevalent social norms regarding maternal and child health behaviors.

### Data management

Survey data were collected using mini-laptops, employing a user-written computer program in CSPro (v6.0) with built-in validation checks. The program was designed to account for all skip patterns and ensure required questions were answered before proceeding. The data collected were reviewed by field supervisors to ensure accuracy and completeness. Data were checked weekly by a co-investigator in Delhi, India.

### Measures

#### Socio-demographic characteristics

The socio-demographic characteristics considered in this paper are based on a single response question capturing a respondent’s age, parity, caste, formal education, engagement in some occupation, and duration of SHG association. Formal education was defined as the ability to both read and write.

#### Exposure to the intervention

Exposure to the intervention was defined as women belonging to SHGs where health messages related to correct maternal and child health outcomes were delivered in at least one weekly group meeting per month.

#### Outcome variables

For this paper, healthy ANC practices were assessed by selecting nine outcome indicators that substantiate the benefits of combining maternal health programs and SHGs, namely: at least four ANC visits, IFA tablet or syrup regimen for at least 100 days, two TT injections, complete ANC (at least four ANC visits, two TT injections, IFA supplements for 100 or more days), ANC accompaniment by SHG members, information from community members (SHG members, CMs) on signs of pregnancy and delivery complications, treatment for pregnancy complications,^1^ supplementary food from *Anganwadi* center, and planning for place of delivery during pregnancy.

### Statistical analyses

Bivariate analyses explored select socio-demographic characteristics of women who belonged to the exposure group and those who belonged to the unexposed group. Chi-square tests checked the association of socio-demographic characteristics and exposure to health intervention. Differences between means were assessed using the student T-test. All tests were double-sided and a *p*-value of less than 0.05 was considered statistically significant.

The radius caliper method of propensity score matching (PSM) was used to examine the impact of health intervention on ANC outcomes [16–18]. Each woman exposed to the health intervention was matched with an unexposed woman whose propensity score fell in a pre-defined neighborhood of the estimated propensity score of the exposed. All socio-demographic variables, namely age, parity, caste, formal schooling, employment, and duration of SHG membership, were used to calculate the propensity score. The propensity score was calculated using logistic regression, which had a dichotomous independent variable of interest, i.e. exposure to the intervention (where 1 = woman who belonged to SHG + H group and 0 = woman who belonged to SHG only group) and observed socio-demographic characteristics as predictor variables.

The key assumption in the PSM approach was based on the propensity score where assignments to exposed and unexposed groups were considered random [19]. One test of this assumption was to examine the balancing property, which states that after matching the distribution of confounding factors is similar among exposed and matched unexposed groups [20]. To examine whether this balancing property was satisfied, differences in the socio-demographic characteristics of exposed women were compared to unexposed women, before and after matching, using the chi-square test (for percentages) and unpaired t-test (for mean values). The overall covariate imbalance of the model was examined by testing the joint significance of all the regressors (i.e. the ability of covariates to predict exposure to any intervention) using the likelihood ratio test before and after matching.

The difference in ANC outcomes between exposed and control groups was directly compared to show the impact of health BCC in the exposed group, known as the average treatment effect on treated (ATT). Additionally, comparing the difference in ANC outcomes between control and matched exposed groups showed the impact of exposure on the unexposed, known as the average treatment effect on the untreated (ATU) [21]. These two average effects were weighted by the proportion of SHG women in exposed and control groups, respectively, to arrive at the impact of the intervention exposure on outcomes, known as average treatment effect (ATE). Data were analyzed using STATA 11.0.

### Ethical considerations

The evaluating institution’s review board (IRB) reviewed and approved the study design and survey questionnaires. A comprehensive informed consent process was followed, with respondents informed about the study, including the interview’s duration (approximately 45 min), and any queries addressed before written consent was requested. In cases where respondents were illiterate or did not want to sign a consent form, verbal consent was taken. A copy of the written consent was provided to respondents, for their records. Participants were not given any monetary compensation for their time.

## Results

### Socio-demographic characteristics and exposure to health intervention

A description of the socio-demographic characteristics of the sample is presented in Table 1. The mean age of respondents was about 27 years. Nearly 68% of SHG women had three or more children, and most (91.5%) were SC/ST. Around one fourth (25.2%) of surveyed SHG women had a formal education, and nearly two-fifths (39.1%) were engaged in some occupation. About 60% of women reported an association with an SHG for 25 or more months, 28% for 13 to 24 months, and nearly 12% for 1 year or less (Table 1).

**Table 1:** Socio-demographic characteristics and exposure to health intervention among women who are part of self-help groups, Bihar, India, 2016 (*N* = 1204)

Socio-demographic characteristics	Women who belong to self-help groups			
	Overall percentage and mean	Exposed to health intervention	Unexposed	*p*-value ^c^
	(*N* = 1204)	(*N* = 597)	(*N* = 607)	
Mean age in years (SD)	26.8 (4.9)	27.5 (5.0)	26.1 (4.7)	< 0.001***
Had 3 or more children	68.2	77.4	59.1	< 0.001***
Belongs to SC/ST ^a^	91.5	91.6	91.4	0.905
Formal schooling ^b^	25.2	17.9	32.3	< 0.001***
SHG women engaged in some occupation	39.1	43.7	34.6	< 0.001***
Duration of association with SHG				
Less than equal to 12 months	11.7	3.5	19.8	< 0.001***
13–24 months	28.2	19.3	37.1	< 0.001***
25 or more months	60.1	77.2	43.1	< 0.001***

SD standard deviation

*p < 0.10; **p < 0.05; ***p < 0.01

^a^ *SC* Scheduled Caste, *ST* Scheduled Tribe

^b^ Formal schooling refers to the ability to both read and write

^c^ *p* values were obtained by comparing percentages for those who were exposed to health intervention and those who were not exposed to health intervention. Differences in percentages were tested using *χ*^*2*^ test statistics and differences in average values were tested using unpaired t statistic

SHG women exposed to the health intervention differed significantly from all unexposed SHG women in almost all socio-demographic characteristics (Table 1), but no significant differences were found when SHG women exposed to the health intervention were compared with matched unexposed SHG women (Table 2). For example, the percentage of women engaged in some occupation in the two groups was highly significant (*p*-value < 0.001) before matching, but after matching they were not significantly different (p-value 0.212). The likelihood ratio Chi-square statistic computed to test the joint significance of covariates reduced significantly after matching among SHG women (before matching: *χ*^*2*^ statistic = 210.7, *p*-value < 0.001; after matching: *χ*^*2*^ statistic = 2.01, p-value 0.959).

**Table 2:** Matching socio-demographic characteristics of SHG women who were not exposed to health intervention with those who were exposed, Bihar, India, 2016

Women who belong to self-help groups			
Socio-demographic characteristics	Exposed to health intervention	Matched unexposed	*p*-value ^d^
Mean age in years (SD)	27.5 (4.6)	27.4 (4.4)	0.917
Had 3 or more children	77.4	77.9	0.831
Belongs to SC/ST ^a^	91.6	90.6	0.540
Formal schooling ^b^	17.9	18.6	0.771
SHG women engaged in some occupation	43.7	40.1	0.212
Duration of association with SHG			
Less than equal to 12 months	3.5	3.7	0.896
13–24 months	19.3	19.3	0.992
25 or more months	77.2	77.0	0.947
N ^c^	597	598	
Likelihood ratio χ2 test statistic (*p* value) ^e^			
Before matching	210.69 (< 0.001)		
After matching	2.01 (0.959)		

SD standard deviation

*p < 0.10; **p < 0.05; ***p < 0.01

^a^ SC-Scheduled Caste, ST-Scheduled Tribe

^b^ Formal schooling refers to the ability to both read and write

^c^ Differences in N values for matched unexposed and unexposed is the number of unexposed cases dropped from the analysis as no match could be found for them

^d^ *p* values were obtained by comparing percentages for those who were exposed to health intervention and matched unexposed SHG women. Matched unexposed refers to SHG women who were unexposed to health intervention and had propensity scores similar to that for those exposed SHG women. Differences in percentages were tested using *χ*^*2*^ test statistics and differences in average values were tested using unpaired t statistic

^e^ The likelihood ratio *χ*^*2*^ test statistic was used to test the joint significance of all the regressors before and after matching

### Impact of health intervention on ANC outcomes

The impact of health intervention exposure on ANC behaviors in matched samples of exposed and unexposed SHG women is presented in Table 3.

**Table 3:** Estimated impact of exposure to health interventions on antenatal care-related outcomes among self-help group women, Bihar, India, 2016

Health behavior outcomes	After matching		Average effect of exposure among exposed (ATT ^e^)			Expected increase in health behavior (ATE ^f^)		
	Exposed %	Unexposed %	ATT	95% CI for ATT	p-value	ATE	95% CI for ATE	*p*-value
Had 4 or more antenatal care visits	15.8	10.8	5.0	(−0.8, 10.8)	0.09	7.2	(0.76, 13.7) **	0.03
Took iron folic acid tablets/syrup for at least 100 days	14.9	7.5	7.4	(0.1, 14.5) **	0.04	8.7	(5.0, 12.5) ***	< 0.001
Received 2 TT injections	77.3	83.9	−6.6	(−10.2, −2.9) ***	< 0.001	−4.8	(− 10.1, 0.5)	0.07
Received full ANC ^a^	4.2	1.6	2.6	(0.3, 4.9) **	0.03	3.6	(2.3, 4.9) ***	< 0.001
Accompanied by SHG members for ANC visits	27.9	13.6	14.2	(10.9, 17.5) ***	< 0.001	13.0	(7.2,18.9) ***	< 0.001
Received information on signs of pregnancy and delivery complications from community members ^b^	46.0	28.0	18.0	(12.6, 23.3) ***	< 0.001	20.9	(11.5, 30.3) ***	< 0.001
Sought treatment for pregnancy complications ^c^	27.5	23.5	4.0	(−0.5, 8.5)	0.08	2.4	(−3.2, 7.9)	0.40
Received supplementary food from Anganwadi center	54.2	51.9	2.3	(−0.2, 4.8)	0.26	2.7	(0.5, 4.9) **	0.01
Planned for place of delivery during pregnancy ^d^	94.5	88.3	6.2	(1.2, 11.1) **	0.01	6.5	(2.4, 10.6) ***	< 0.001

*p < 0.10; **p < 0.05; ***p < 0.01

^a^ Full ANC is defined as receiving 4 or more antenatal care visits, 2 TT injections and consumption of 100 or more iron-folic acid tablets during pregnancy

^b^ Information on signs of pregnancy and delivery complications such as swelling of hands, paleness, weakness, visual disturbances, excessive fatigue, weak or no movement of fetus, abnormal position of fetus, excessive vomiting, hypertension, jaundice, excessive bleeding, convulsions, prolonged labor, obstructed labor, foul-smelling discharge, delay in placental expulsion or retained placenta, cord prolapsed/ baby’s hand and feet coming out first and cord around the neck of the baby from community members like SHG leader, SHG members, Saheli or Community Mobilizer

^c^ Severe pregnancy-related complication such as weak or no movement of the fetus, hypertension/ high BP, jaundice, excessive bleeding and convulsions

^d^ Prior to delivery planned to deliver the child at home or in a healthcare facility

^e^ Average treatment effect for the treated (ATT)/ exposed SHG women measured the impact of the intervention on exposed SHG women

^f^ Average treatment effect (ATE) represents the impact of the program obtained by averaging the impact across all the individuals (exposed and unexposed

Results suggest that exposure to the health intervention increased the likelihood of: 1) at least four ANC visits among SHG women (ATE = 7.2, 95% CI: 0.76–13.7, *p*-value < 0.05), 2) consumption of IFA for at least 100 days (ATE = 8.7, 95% CI: 5.0–12.5, p-value < 0.001), and 3) complete ANC (ATE = 3.6, 95% CI: 2.3–4.9, p-value < 0.001), when compared to women not exposed to the health intervention. Exposure to health intervention also showed an increase in other related ANC outcomes such as ANC accompaniment by SHG members, information on signs of pregnancy and delivery complications, and delivery planning (Table 3). Exposure to health intervention was not found to have impacts on two TT injections or seeking treatment for pregnancy-related complications among SHG members.

## Discussion

The findings from this study, following an intervention that provided information on correct ANC to women in SHGs, reveal significant increases in most ANC outcomes within 1 year of the intervention, compared to unexposed matched SHG women, after controlling for respondents’ socio-demographic characteristics. This is consistent with similar research on the effects of SHG interventions, demonstrating that combining a health program with microfinance-based SHG activities is associated with significant increases in maternal care services [12, 22]. As evident from other studies conducted in Nepal and Maharashtra, leveraging microfinance-based SHGs to promote health outcomes is beneficial to the health and well-being of their members [23]. This study adds to existing evidence by drawing attention to the significant improvement in ANC practices due to a scaled-up intervention focused among the most marginalized populations.

Improvements in several ANC practices can be explained by an increase in SHG members reporting accompaniment by fellow group members to ANC visits. This reveals that SHGs are not only a platform to alleviate poverty, but are an efficient and effective model for building social capital through collectivization and cohesion, which play a vital role in the development of marginalized populations [12, 23, 24]. Health information supplied through SHGs provides an added benefit of mutual support among members for ANC service access. Sharing health messages in SHG meetings changed the normative practice of ANC in these communities, benefiting highly marginalized women. As a result of this changing environment, frontline workers may also become more proactive in following up with pregnant women for ANC service and providing them with timely care throughout pregnancy.

This study did not find any difference in either group of women for the treatment of pregnancy complications. As recognized by studies in other low- and middle-income countries, pregnant women do not seek treatment due to factors ranging from lack of information, financial constraints, multiparity, in addition to cultural factors [4, 25]. Focused interventions with male involvement have shown some results in improved health-seeking behavior of pregnant women, which was not the focus on this intervention [26–28]. Further research is needed in Bihar to identify how efforts can be made to increase treatment-seeking for pregnancy complications by empowering women to seek care, increasing their financial independence, for better health service access, and engaging family members, in case of a health emergency. No differences in both groups were found for TT injections either, which could be due in part to interrupted or irregular injection supplies.

For the ANC outcomes that did not show changes as expected, in the group that received health messaging, periodic reviews of meeting and messaging content, quality of message conveyance, and frequency of delivery in various locations may be helpful [29]. In addition to examining individual behaviors, any challenges CMs faced in delivering health messages should be addressed.

While these findings provide important insights on the effect of sharing health messages with SHG members, they must be interpreted in relation to certain study limitations. First, the outcome indicators for ANC were based on self-response, which is susceptible to both social desirability and recall bias. The potential for recall bias was reduced by recruiting trained and experienced enumerators for the interviews, and by selecting mothers who had a live birth in the 12 months preceding the survey. Secondly, the findings are specific to eight blocks from selected districts of Bihar and cannot be generalized to other states. Replication of this study in other settings requires further research. Lastly, PSM relies on the conditional independence assumption, that is, all available variables considered to be a predictor of exposure to health intervention are included in the model used to estimate propensity scores. It is possible that some potential predictors of exposure to the health intervention were not available from our study, such as wealth index or frequency of contact with frontline workers, and therefore could not be accounted for in the analysis.

## Conclusions

These study findings reiterate that health messaging through existing microfinance-based SHGs is a viable approach for improved ANC practice. Exposure to health messages within SHGs is associated with an increased likelihood of at least four ANC visits among SHG women, IFA consumption for at least 100 days, and complete ANC when compared to SHG women not exposed to the health intervention. This approach helps reduce current deficiencies in information on correct ANC practices while increasing the demand for health services and encouraging pregnant women to seek ANC from healthcare providers.

With nearly seven million households involved in SHGs organized by JEEViKA, and more expected to join due to the support of important state authorities, SHGs have the potential to increase social capital and reach among the most marginalized populations at an unprecedented scale. This health BCC intervention could be scaled up throughout Bihar in a phased manner. Health messages can also be individuated to specific groups’ or members’ needs, through systematic training and capacity-building of CMs, who are responsible for delivering the health modules.

Periodic observations, monitoring, reviews, and recommendations would strengthen the program’s internal quality and monitoring. Further research is needed to measure the sustainability of this integrated approach and identify optimal program frequency, for continuous improvements in health outcomes. Exploring the diffusion of health messages through wider communities, beyond those families with direct SHG involvement, and its impact on targeted practices apart from SHG members and their families would be worthwhile as well.

## Data Availability

The dataset used and/or analyzed during the current study are available from the following link: [link retracted to ensure anonymity].

## Abbreviations

ANC: Antenatal care
ATE: Average treatment effect
ATT: Average treatment effect on treated
ATU: Average treatment effect on untreated
CM: Community mobilizer
IFA: Iron folic acid
MMR: Maternal mortality ratio
PSM: Propensity score matching
SC/ST: Scheduled castes/ scheduled tribes
SHG: Self-help group
TT: Tetanus toxoid
WHO: World Health Organization

## Footnotes

1 Severe pregnancy related complication include weak or no movement of fetus, hypertension/ high BP, jaundice, excessive bleeding and convulsions
